# Supplementary material for: Temperature Differentially Affects Gene Expression in Antarctic Thraustochytrid Oblongichytrium sp. RT2316-13
Source: Mar Drugs. 2020 Nov 18;18(11):563. doi: 10.3390/md18110563 (PMC7698632; doi:10.3390/md18110563)
Supplement: Supplementary file 1 [file marinedrugs-18-00563-s001.zip › marinedrugs-18-00563-s001/CShene_Supplemental Material_2.pdf]

Supplemental Material

Temperature differentially affects gene expression in Antarctic thraustochytrid *Oblongichytrium* sp. RT2316-13

Paris Paredes, Giovanni Larama, Liset Flores, Allison Leyton, Carmen Gloria Ili, Juan A. Asenjo, Yusuf Chisti, Carolina Shene

1. Table S1 to S3.
2. Figure S1.
3. Excel file Paredes_et_al.xlsx.

Table S1. Sequencing results. The initial amount of reads (total reads) and the number of reads that passed quality control filters (HQ reads).

**Condition**	**ID**	**Total reads**	**HQ reads**	**HQ reads (%)**
Control	GM1595-1	30,853,728	30,688,242	99.46
Control	GM1595-2	47,592,052	47,358,355	99.51
Control	GM1595-3	21,876,024	21,759,224	99.47
Treatment	GM1595-4	25,468,998	25,337,912	99.49
Treatment	GM1595-5	24,655,850	24,529,757	99.49
Treatment	GM1595-6	24,124,200	23,985,768	99.43

Table S2. Total number of high quality (HQ) reads that were mapped to a sequence in RT2316-13 transcriptome, concordantly one or more times (properly aligned), and the total number of reads aligned (overall aligned), with their respective mapping rate (%).

ID	HQ reads	Properly aligned	Overall aligned	Properly aligned mapping rate (%)	Overall mapping rate (%)
GM1595-1	30,688,242	27,437,849	30,383,019	89.41	99.01
GM1595-2	47,358,355	42,773,627	46,999,661	90.32	99.24
GM1595-3	21,759,224	19,310,940	21,553,763	88.75	99.06
GM1595-4	25,337,912	24,353,793	25,094,881	96.12	99.04
GM1595-5	24,529,757	23,699,786	24,327,830	96.61	99.18
GM1595-6	23,985,768	21,237,876	23,773,688	88.55	99.12
			Average	91.63	>99.0

Table S3. Primers used for identifying genes related to production of fatty acids.

Gene	Gene abbreviation[§]	Sequence	T_m (℃)
Malic enzyme	Me_F	AAGCCTGCCAAGAGTTCCA	56.9
	Me_R	GCCAGTTGTTCACGAGAGTC	55.9
Acetyl-CoA carboxylase	ACC_F	GAGACCACTTACCGCCTGTT	57.0
	ACC_R	CGCCAATGAGCACAAGGAAG	56.8
PKS subunit A	Pfaa_F	AGCCTCCTTGATAGCCTTCTC	56.2
	Pfaa_R	TCTGGTGCGTGTTCTTGGT	56.9
PKS subunit C	Pfac_F	CTGGTGGTGGTGTTGGATG	56.1
	Pfac_R	GCTGCTTGCGGACATTGT	56.6
18S rRNA	18s_F	TGCCGACTTGCGATTGTTG	56.4
	18s_R	TTCAGCCTTGCGACCATACT	56.5

[§]Forward primers (_F) and reverse primers (_R). The sequences were obtained from Ma et al. [29]. T_m = melting temperature.

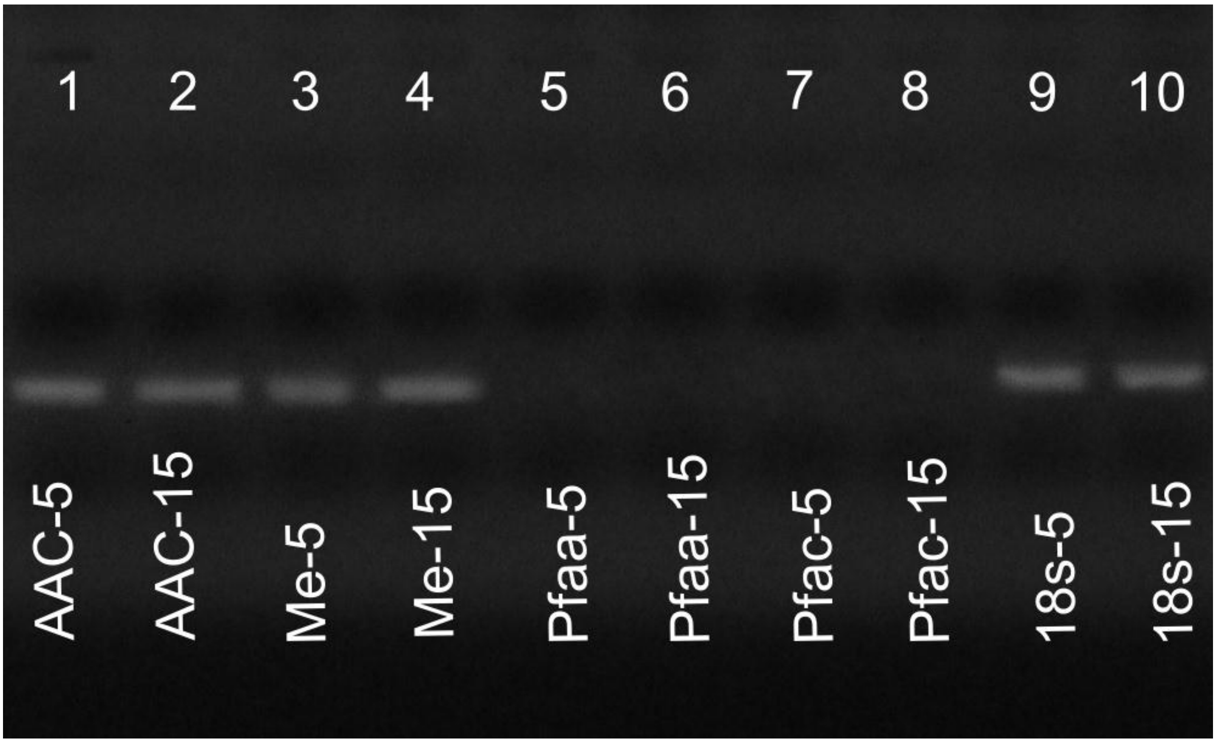

Figure S1. Amplification of cDNA segments. AAC, acetyl-CoA carboxylase; Me, malic enzyme; Pfaa, subunit A of PKS gene; Pfac, subunit C of PKS gene; 18s, 18S subunit of RNA. xx-5 and xx-15 denote cDNA of cells grown at 5 and 15 ℃, respectively.
